# Estimating the efficacy of community-wide use of systemic insecticides in dogs to control zoonotic visceral leishmaniasis: A modelling study in a Brazilian scenario

**DOI:** 10.1371/journal.pntd.0006797

**Published:** 2018-09-17

**Authors:** Sonia A. Gomez, Lloyd A. C. Chapman, Erin Dilger, Orin Courtenay, Albert Picado

**Affiliations:** 1 ISGlobal-Hospital Clínic, Universitat de Barcelona, Barcelona, Spain; 2 Department of Global Health and Development, London School of Hygiene and Tropical Medicine, London, United Kingdom; 3 School of Life Sciences, University of Warwick, Gibbet Hill Campus, Coventry, United Kingdom; RTI International, UNITED STATES

## Abstract

Systemic insecticides in dogs have been suggested as a public health intervention to prevent human cases of Zoonotic Visceral Leishmaniasis (ZVL). But, currently there are no systemic insecticides for dogs registered against zoo-anthropophilic pool blood feeding phlebotomine flies. We predict the impact of community-wide use of systemic insecticide in dog populations as a public health measure to control transmission of *Leishmania infantum* to humans using a mathematical model. We developed a Susceptible-Exposed-Infected (SEI) compartmental model to describe *L*. *infantum* transmission dynamics in dogs, with a vectorial capacity term to represent transmission between *L*. *infantum-*hosting dogs via phlebotomine flies. For Infected (I) dogs two levels of infectiousness were modelled, high infectiousness and low infectiousness. Human incidence was estimated through its relationship to infection in the dog population. We evaluated outcomes from a wide range of scenarios comprising different combinations of initial insecticide efficacy, duration of insecticide efficacy over time, and proportion of the dog population treated (60%, 70% & 80%). The same reduction in human infection incidence can be achieved via different combinations of insecticide efficacy, duration and dog coverage. For example, a systemic insecticide with an initial efficacy of 80% and 6 months above 65% efficacy would require treating at least 70% of the dogs to reduce the human infection incidence by 50%. Sensitivity analysis showed that the model outcome was most sensitive to baseline values of phlebotomine fly daily survival rate and insecticide coverage. Community-wide use of systemic insecticides applied to the “*L*. *infantum* canine reservoir” can significantly reduce human incidence of *L*. *infantum* infection. The results of this mathematical model can help defining the insecticide target product profile and how the insecticide should be applied to maximise effectiveness.

## Author summary

Zoonotic visceral leishmaniasis (ZVL) is a potentially deadly disease in humans caused by *Leishmania infantum*. This leishmania species can be delivered by pool blood feeding zoo-anthropophilic phlebotomine flies to several mammals, the dog population being recognized as the main reservoir. Transmission from infected dogs to humans is through the bite of female phlebotomine sand flies. The disease is endemic in several countries and Brazil has a high prevalence of cases with over 3000 ZVL cases reported per year. The main, inefficient and highly controversial, control measure in Brazil has been culling sero-positive dogs. The community-wide use of systemic insecticides in dogs could be an alternative to control *L*. *infantum* transmission from phlebotomine flies to humans. The rationale is that phlebotomine flies which sampled their blood meals from dogs treated with systemic insecticides would die reducing the risk of *L*. *infantum* transmission. To reduce the number of ZVL cases, a large proportion of dogs in the community should be treated and the systemic insecticide used should be effective in killing phlebotomine flies acting as vectors of *L*. *infantum* parasites for a significant amount of time. We used a mathematical model mimicking *L*. *infantum* transmission to show that this novel vector control strategy could be effective. We identified the combination of different key parameters (e.g. insecticide efficacy, duration and proportion of dogs treated) that could lead to a significant reduction of the risk of *L*. *infantum* infection in humans.

## Introduction

The protozoan parasite *Leishmania infantum* is the etiological agent of Zoonotic Visceral Leishmaniasis (ZVL) in humans and dogs. This pathogen can also infect other mammals, but dogs are the main reservoir causing human infections [1–3]. Transmission of *L*. *infantum* to humans occurs through the bite of female phlebotomine sand flies previously infected by biting infected dogs [4, 5], whereas humans are not considered a reservoir of *L*. *infantum* [6–8]. ZVL in humans is characterized by fever, weight loss, hepato- and spleno-megaly, and anemia [9], and the fatality rate can be very high if untreated [9,10]. The reported case numbers of human ZVL in Brazil has persisted above 3000 cases per year since 1994 despite intervention policies of reservoir reduction and sand fly control against transmission [11]. Indeed, since the 1980s, endemic transmission has expanded into more urban and peri-urban areas, beyond the historic predominantly rural transmission foci [12–14]. In endemic areas of ZVL and particularly in Brazil, a national policy of test-and-slaughter of sero-positive dogs has been the main control strategy, though this method continues to be highly controversial [15–17]. Additional control measures include early diagnosis and treatment of human cases, and reactive chemical control of the vector [11]. Despite these combined efforts, ZVL transmission continues to expand in Brazil [18,19].

A proven method to reduce *L*. *infantum* transmission is by insecticide-impregnated collars applied to dogs [20–22]. Community-wide deployment of deltamethrin-impregnated collars has proven also to reduce human infections incidence with *L*. *infantum* [23]. However, the cost of the collars, their high loss rate (requiring continual surveillance and replacement) and the logistics required to deploy them at a mass scale limit their use as a public health intervention in endemic regions [24,25]. Systemic insecticides could be an alternative to impregnated collars and their community-wide use in dogs may control *L*. *infantum* infection in humans in endemic areas [26]. In theory mass treating dogs with systemic insecticides may be easier than deploying impregnated collars. Oral treatments (e.g. treated baits [27] or chewable tablets [28,29]) could be used to significantly reduce dog handling.

Currently there are no systemic insecticides for dogs registered against sand flies but the effect of mass drug administration of drugs with an insecticidal effect has already being evaluated on anthroponotic VL [30] and malaria [31], with mathematical models also providing further support for their use in these cases [32,33]. Mathematical modelling has similarly been used to estimate the efficacy of control strategies for ZVL [34], specifically the culling of sero-positive dogs [15,16,35] or the use of insecticide impregnated collars or vaccines [16,36]. Recent and more complex models have aimed to better understand and predict *L*. *infantum* transmission dynamics [37,38].

The aim of this study is to evaluate the efficacy of community-wide use of systemic insecticides in dogs as a strategy to reduce the number of human infections with *L*. *infantum* causing ZVL cases in an endemic area using a parsimonious deterministic mathematical model. The modelling exercise will also help defining the minimum requirements for developing systemic insecticides for dogs against sand flies.

## Methods

**Fig 1:**
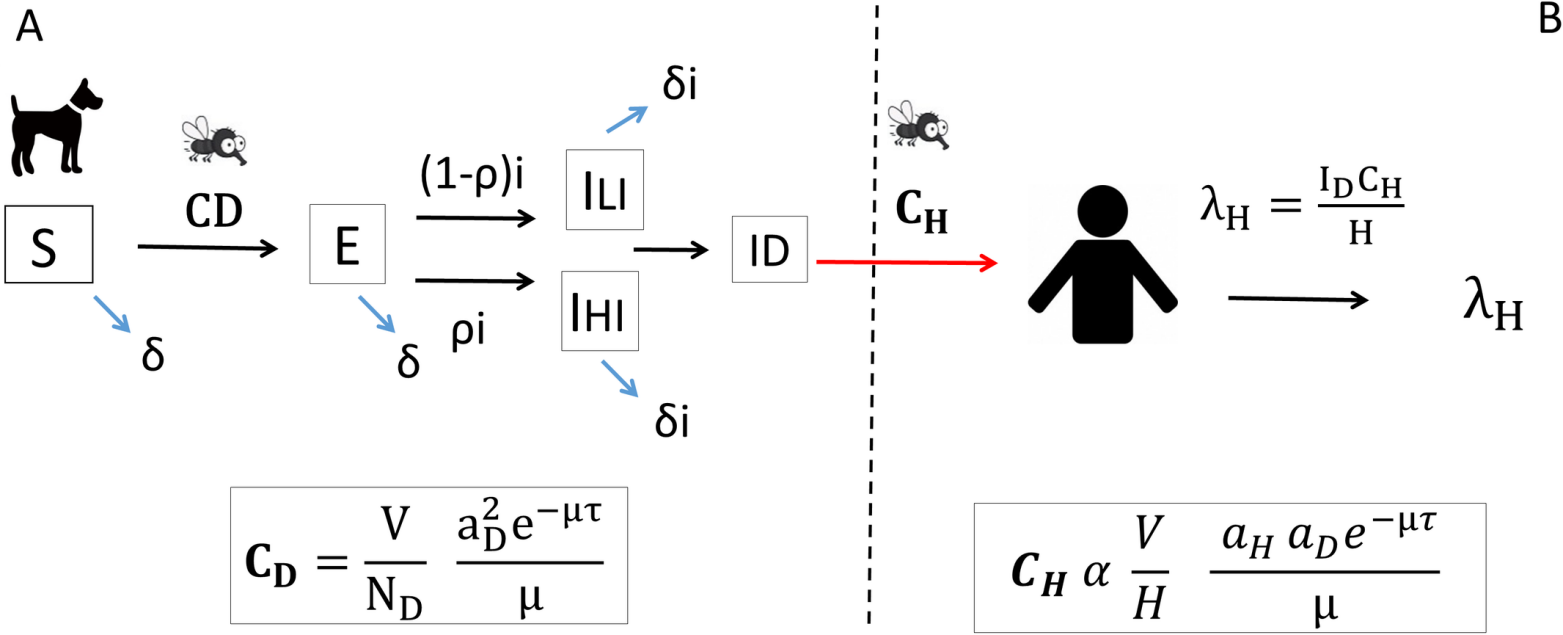
Model representing the transmission dynamics of *L*. *infantum*. (A) Compartmental model to calculate transmission between dogs: Susceptible (S)–Exposed (E)—Infectious (I). Proportion ρ of E dogs become highly infectious (I_HI_), and 1-ρ become low infectious (I_LI_). Vectorial capacity (C_D_) represents the transmission of *L*. *infantum* among dogs. (B) Equations to estimate transmission from infected dogs to humans in the form of human incidence of ZVL (λ_H_). Vectorial capacity (C_H_) represents the transmission of *L*. *infantum* from dogs to humans. All the parameters included in C_D_, C_H_ and λ_H_ are defined in Table 1.

The transmission dynamics of *L*. *infantum* was modelled assuming that only infected dogs are capable of infecting sand flies, i.e. assuming that infected people do not contribute significantly to transmission relative to dogs (Fig 1). To model the transmission dynamics, we used (i) a deterministic mathematical model to calculate transmission to dogs, and (ii) a set of equations to estimate transmission from infected dogs to humans, extended and developed from Dye (1996) [39]. Using the deterministic model, we simulated different intervention scenarios and calculated the number of infected dogs in the population for each scenario, and then estimated the number of new human infections arising from transmission from the infected dogs.

### Transmission to dogs

We used a Susceptible-Exposed-Infectious (SEI) compartmental model to describe the transmission dynamics of ZVL in dogs (Fig 1), where susceptible (S) dogs become exposed (E) after being bitten by an infected sand fly, and after an exponentially distributed incubation period (with average duration 1/i, where i is the incubation rate per day) become either highly infectious (I_HI_) or low-infectious (I_LI_) [15], whereupon they can infect other dogs via the vector. The model uses a vectorial capacity term (C_D_) to represent the transmission of *L*. *infantum* between dogs by sand flies. This approach is appropriate because the infection dynamics happen on a much faster time scale in sand flies than in dogs, and few sand flies live long enough to acquire infection. The formula for C_D_ (1) includes the following terms: number of sand flies (V), number of dogs (N), biting rate on dogs (a_D_), sand fly mortality rate (μ), and probability of surviving the fixed extrinsic incubation period τ (e^−μτ^)

$$C_{D}=\frac{V}{N}\frac{a_{D}^{2}e^{-\mu \tau }}{\mu },$$ (1)

Exposed (E) dogs represent recently infected dogs that do not transmit infection and do not show clinical symptoms. The fraction of dogs that become highly infectious (I_HI_) is denoted ρ, so (1 − ρ) become low infectious (I_LI_). The set of differential equations that describe the dynamics in a stable population are:

$$B=\delta \;(S{+E)+\delta_{i}(I}_{LI}+I_{HI}),$$ (2)

$$\frac{dS}{dt}=B-p_{D}p_{v}^{li}\frac{C_{D}SI_{LI}}{N}-p_{D}p_{v}^{hi}\frac{C_{D}SI_{HI}}{N}-\delta S,$$ (3)

$$\frac{dE}{dt}=p_{D}p_{v}^{li}\frac{C_{D}SI_{LI}}{N}+p_{D}p_{v}^{hi}\frac{C_{D}SI_{HI}}{N}-(i+\delta )E,$$ (4)

$$\frac{dI_{HI}}{dt}=\rho iE-{\delta_{i}I}_{HI},$$ (5)

$$\frac{dI_{LI}}{dt}=(1-\rho )iE-\delta_{i}I_{LI},$$ (6)

$$N=S{+E+I}_{LI}+I_{HI},$$ (7)

All terms and values used in Eqs (2)–(7) are described in Table 1. The system of differential Eqs (1)–(7) were solved using the package deSolve in R 3.2.0 [40].

**Table 1:** Parameters in the model and their sources.

Parameter	Definition	Value	Reference
ρ	Proportion of highly infectious dogs	0.17	[15]
i	Incubation rate in dogs	0.005/day	[41]
δ	Death rate in non-infectious dogs	0.0011/day	[15]
δ_i_	Death rate of infected dogs	0.003006/day	[42]
a_D_	Biting rate on dogs	0.333/day	[43]
a_H_	Biting rate on humans	0.125/day	[36]
τ	Latent period of *L*. *infantum* in sand flies	7 days	[39]
μ	Sand fly mortality rate	0.42/day (57%)	[39]
μ_T_	Sand fly mortality rate under treatment	Variable (57–100%)	-
V	Number of sand flies	12000	Fixed
H	Number of humans	1000	Fixed
N	Number of dogs	1000	Fixed
$p_{v}^{hi}$	Probability of a highly infectious dog transmitting to a sand fly	0.39	[15]
$p_{v}^{li}$	Probability of a low-infectious dog transmitting to a sand fly	0.017	[15]
p_D_	Probability of an infected sand fly transmitting to a dog	0.321	[16]
*P*_*T*_	Proportions of dogs treated with systemic insecticides	Variable (60, 70, 80%)	-
Δ	Insecticide decay/day in insecticide efficacy after the administration	Variable(-0.0001, -0.05)	-

### Transmission to humans

Human infection incidence (λ_H_) is related to the number of infected dogs (*I*_*D*_ = *I*_*HI*_ + *I*_*LI*_) and to the capacity of sand flies to transmit to humans (C_H_) [39]

$$\lambda_{H}=\frac{I_{D}C_{H}}{H},$$ (8)

$$C_{H}=\frac{V}{H}\frac{a_{H}a_{D}e^{-\mu \tau }}{\mu },$$ (9)

The *per capita* human incidence rate amongst the susceptible population (λ_H_) was calculated using Eqs (8) and (9).

### Parameter values

Model parameter values (Table 1) were largely obtained from a cohort study of naturally infected Brazilian dogs under high transmission [15,41,44]. In this setting it was shown that a small fraction (17%) of infected dogs were highly infectious, being responsible for 80% of all transmission events measured by longitudinal xenodiagnosis [15]. Therefore, our model included two types of infected dogs: highly infectious (I_HI_) and low-infectious (I_LI_) dogs. For the highly infectious dogs the probability of transmitting infection ($p_{v}^{hi}$ = 0.39) was much higher than for the low-infectious dogs ($p_{v}^{li}$ = 0.017) [15]. The following fixed values were used for the number of sand flies (V = 12000), number of dogs (N = 1000), and number of humans (H = 1000) as these were the parameters for which the model reached equilibrium at 0.02 to 0.03 *L*. *infantum* infections/1000 susceptible people. This is the incidence reported in endemic areas in Brazil [11].

**Fig 2:**
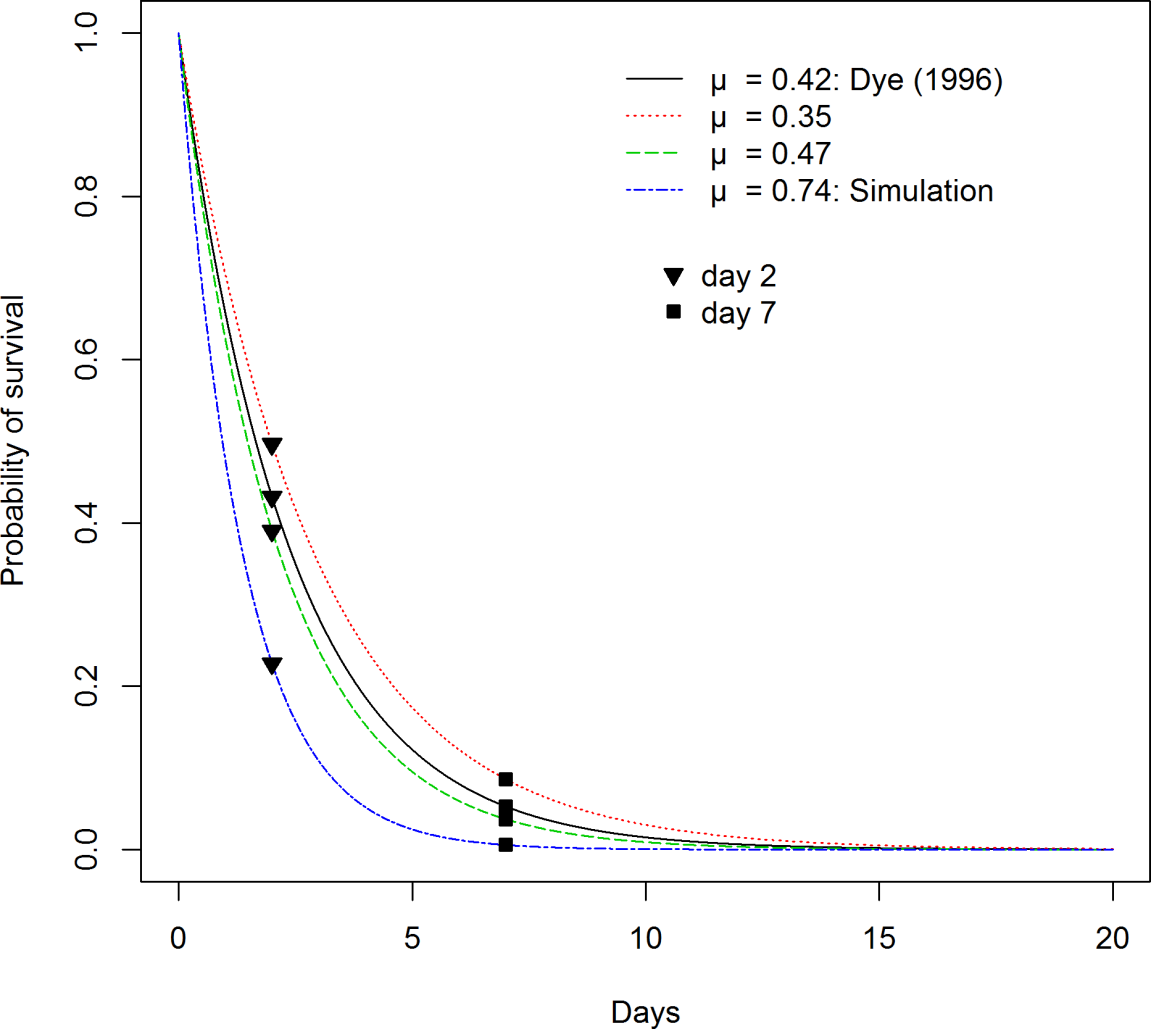
Sand fly survival curve showing the continuous probability of sand fly survival. Blue dashed-dotted line represents the sand fly survival after biting a dog treated with a systemic insecticide of 80% efficacy where only 20% of the sand flies survive after 2 days (black triangle). Black line is the baseline sand fly mortality reported by Dye, 1996. Red dotted line represents the lower bound used in the sensitivity analysis. Green dashed line represents the upper bound used in the sensitivity analysis. Black triangles represent survival 2 days after biting on dogs. Black squares represent survival of *L*. *infantum* extrinsic incubation period (7 days).

The value chosen for the natural sand fly mortality rate (μ = 0.42) was reported by Dye in 1996 [39]. This parameter was estimated from the parous rate in a study of the aggregation behavior of the South American vector, *Luztomyia longipalpis* where they observed 212 sand flies out of 746 survived one cycle (212/746 = 0.284) [43]. From the parous rate the mortality rate was calculated as μ = −ln (0.284) = 1.26/cycle, and life expectancy of 1/1.26 = 0.79 cycles; given a gonotrophic cycle of 3 days on average the average life expectancy of *Lu*. *longipalpis* is estimated to be 2.4 days (corresponding to a death rate of μ = 0.42/day) equivalent to 57% mortality at day 2 and 95% mortality at day 7 (Fig 2).

### Scenarios for prediction

The sand fly mortality rate under treatment (μ_T_), and the proportion of dogs treated with systemic insecticides (coverage) (P_T_), were varied to make model predictions of the efficacy of the intervention to prevent human infection. For the non-treatment scenario, the natural sand fly mortality rate μ = 0.42/day [39] was used. For the treatment scenarios the sand fly mortality rate was used as a proxy of the insecticide efficacy. Insecticide efficacy was included in the vectorial capacity equation in the term defining the sand fly longevity (e^−μ(t)τ^/μ(t)). The effect of a proportion P_T_ of dogs being treated with systemic insecticide on the overall sand fly mortality rate μ(t) was modelled as:

$$\mu (t)=(1-P_{T})\mu_{U}+P_{T}\mu_{T}(t)$$ (10)

where μ_U_ is the natural sand fly mortality rate (μ_U_ = 0.42/day) from feeding on untreated dogs, and μ_T_(t) is the (time-dependent) mortality rate from feeding on treated dogs (see below).

These intervention parameters were tested in combination, whereby coverage scenarios (P_T_) ranged from 60–80% and insecticide efficacies (lethality) were tested from the minimum of 57%, (equivalent to the natural sand fly mortality (Fig 2)) to a maximum of 100% 2 days after blood feeding on a treated dog. Scenarios also included a decrease in insecticide efficacy over time, reflecting a linear daily rate of decay in insecticide efficacy per day post insecticide administration. For each level of insecticide efficacy we simulated a range of decay scenarios, from negligible decay over time (Δ_*min*_ = 0.0001/day) to rapid decay, eliminating efficacy within 10 days of treatment (Δ_*max*_ = - 0.05/day). All scenarios were run for 365 days (after first running the dynamics to equilibrium). With the slope we estimated the time duration for which the insecticide efficacy is above 65%, the minimum efficacy reported for systemic insecticides [45].

Different combinations of the target parameter values were run to identify the parameter space that resulted in ≥ 50% and ≥ 80% reductions in annual human infection incidence. The percentage reduction in human incidence is given by:

$$\%reduction\;in\;\lambda_{H}=100\;\left(1-\frac{\lambda_{H_{i}}}{\lambda_{H_{0}}}\right),$$ (11)

where $\lambda_{H_{0}}$ is the median equilibrium human incidence calculated from the model run with initial values of 1000 dogs (S = 998, E = 0, I_HI_ = 1, and I_LI_ = 1) and 12000 sand flies, and $\lambda_{H_{i}}$ is the median human incidence during the 365 days of the intervention, calculated from each scenario.

### Sensitivity analysis

*L*. *infantum* transmission models have been reported as being highly sensitive to some of the parameters included in our model [16,37,38]. Univariate sensitivity was performed by selecting biologically realistic lower and upper bounds of the following parameters: biting rate on humans (a_H_), biting rate on dogs (a_D_), natural sand fly mortality rate (μ_U_), sand fly density (V/N), proportion of highly infectious dogs (ρ), probability of an infected sand fly transmitting to a dog (p_D_) and death rate of infected dogs (δ_i_). We also included insecticide coverage (proportion of the dog population treated), P_T_, and decay in insecticide efficacy, Δ, in the sensitivity analysis to observe how these parameters affected model predictions compared to the aforementioned parameter values.

For the purposes of the sensitivity analysis, our outputs at equilibrium were $\lambda_{H_{0}}$, S, E, I_HI_, and I_LI_, and the chosen baseline intervention scenario was 80% coverage, 80% insecticide efficacy and a linear decrease in efficacy of Δ = −0.00128/day (i.e. monthly decay of 4%).

## Results

### Model equilibrium

Running the model for 10,000 days, the equilibrium number of dogs in each infection class were 508 susceptible (S = 508), 130 exposed (E = 130), 62 highly infectious (I_HI_ = 62), and 300 low-infectious (I_LI_ = 300) dogs, and *per capita* human incidence $\lambda_{H_{0}}$ = 0.0227 infections/1000 susceptible people/year.

### Model outcomes

**Fig 3:**
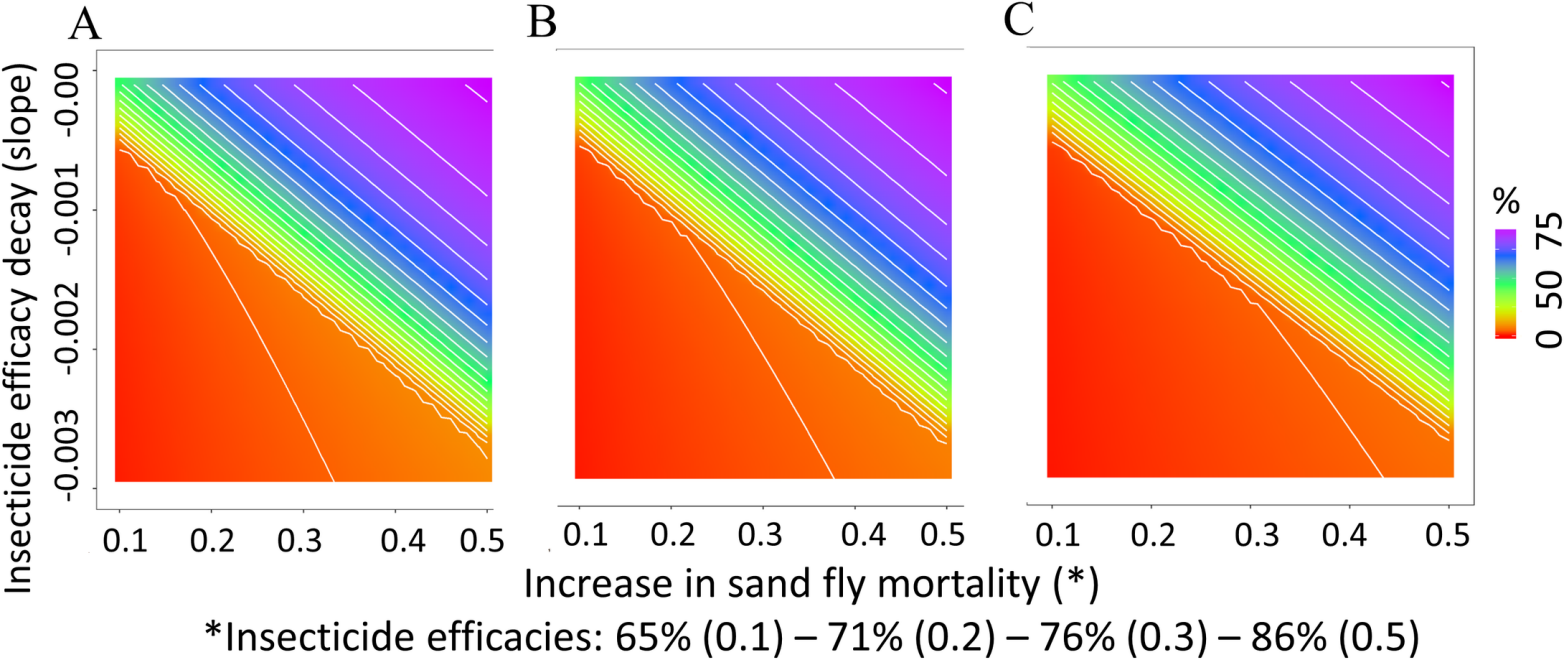
Reduction of human incidence of *L*. *infantum* infection. **Scenario of mass application of systemic insecticides to dogs**. Dog coverage: 80% (A), 70% (B) and 60% (C). Insecticide efficacy (horizontal axis) is represented by the increase in sand fly mortality caused by the insecticide (μ_T_(0)). Decay in the insecticide efficacy occurs at a constant rate per day (vertical axis). Contour curves mark 5% changes in human incidence.

Including combinations of initial insecticide efficacy (57–100%) and efficacy decay (0.001/day—0.05/day), the model predicted reductions of 0 to 97% in human infection incidence when dog population coverage was 80%. At 70% and 60% dog coverage the maximum reductions in human incidence achieved were similarly high, 95% and 93% respectively (Fig 3).

Reductions of ≥ 50% in human incidence were achieved with an initial insecticide efficacy of ≥ 80% and efficacy above 65% maintained for a least 5.7, 6.1, and 6.5 months when under 80%, 70% and 60% dog coverage respectively (Table 2). The model predictions also showed that change in human incidence was most sensitive to variations in the initial levels of insecticide efficacy and efficacy decay or duration once the incidence reduction was between 50 and 75% (contour curves Fig 3).

**Table 2:** Example of identification of combinations of dog coverage, insecticide efficacy and monthly decay in efficacy that lead to 50% and 80% reduction in human incidence according to the model.

Target: reduction in human incidence	Dog coverage (% dogs treated)	Initial Insecticide efficacy (% mortality)	Monthly decay in efficacy	Months with efficacy ≥ *65%
50%	80%	90%	10.6%	5.9
		80%	4.8%	5.7
	70%	90%	10.4%	6
		80%	4.6%	6.1
	60%	90%	10%	6.25
		80%	4.3%	6.5
80%	80%	90%	8.5%	7.4
		80%	2.7%	10.25
	70%	90%	7.9%	7.9
		80%	2.2%	12
	60%	90%	7.2%	8.6
		80%	1.5%	12

*Minimum efficacy reported in systemic insecticide efficacy studies [45]

The model also allows us to estimate the dog population coverage required to reduce human incidence by 50% for an insecticide with given characteristics. For example, a systemic insecticide with an initial efficacy of 80% and 6.5 months above 65% efficacy would require a coverage of 60% to reduce the human infection incidence by 50%. For the same reduction in human incidence an insecticide with 80% initial efficacy and 5.7 months above 65% would require a coverage of 80%.

Testing the ranges of these intervention parameters together, the transmission model allows us to identify all additional combinations of dog coverage, insecticide efficacy and duration that lead to a similar reduction in human incidence. Taking 50% and 80% reduction in human incidence as two significant thresholds, we find a group of combinations (initial efficacies from 80 to 90%, coverage from 60 to 80%, and duration above 65% from 5.7 to 12 months) that will lead to the desired result (Table 2).

### Sensitivity analysis

**Fig 4:**
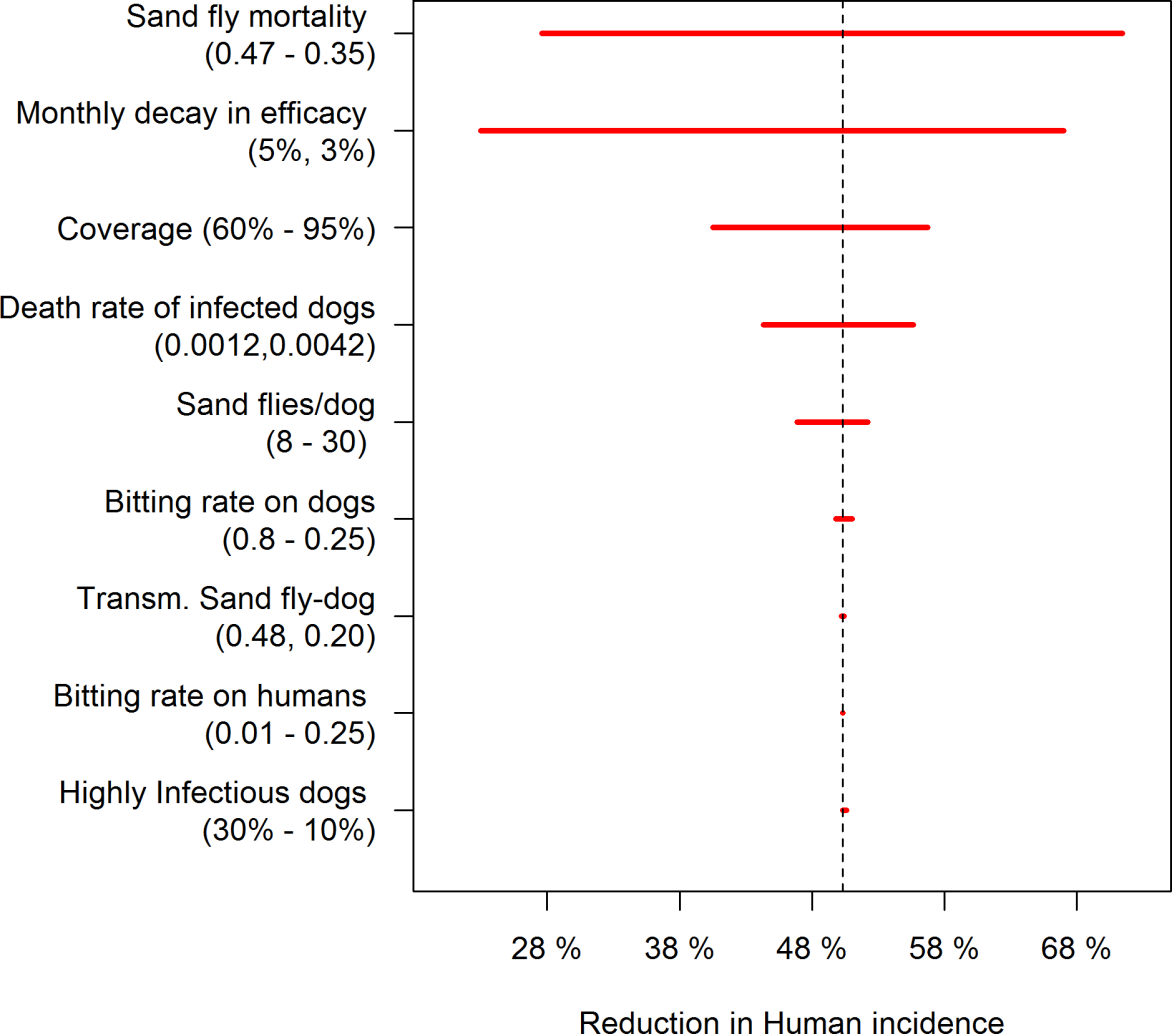
Tornado plot showing the sensitivity of different parameters on the reduction in human incidence of *L*. *infantum* infection in the model.

The estimated reduction in human incidence in the baseline intervention model used in the sensitivity analysis was 50.3%. Of the intervention parameters, model predictions were most influenced by sand fly mortality (Fig 4). A 28% change in sand fly mortality resulted in a 40% change in the reduction in human incidence (Fig 4). It had a greater modification effect than dog coverage, for which a 35% change produced only a 20% change in the estimated incidence reduction. Likewise, a ±25% variation in the decay rate of insecticide efficacy resulted in a -17 and +27% change in human incidence compared to baseline. Lower influence was found in death rate of infected dogs for which a ± 40% change induced a ± 5% change in human incidence (Fig 4).

## Discussion

Using a mathematical model of *L*. *infantum* transmission, we have predicted that significant reductions in human incidence of infection can be achieved by community-wide use of systemic insecticides in dogs.

The model allows estimating the minimum requirements of the systemic insecticide (efficacy and duration) and the intervention (dog treatment coverage) to significantly reduce *L*. *infantum* infections in humans. For example, reducing annual ZVL incidence by 50% would require treating at least 70% of the dogs using an insecticide with an initial efficacy greater than 80% and that would remain effective (mortality over 65%) for at least 6 months. Different combinations of insecticide efficacy, duration and coverage could reach similar impact.

Currently there are no systemic insecticides for dogs registered against phlebotomine sand flies, but some of the existing insecticidal products [26] may comply with some of the requirements identified in our model. In a previous study we showed that fluralaner administered orally to dogs, currently registered for fleas and ticks, had a phlebotomine mortality effect of 60 to 80% for 30 days [46]. The initial insecticide effect may be adequate but its duration seems to be limited for control of ZVL. Slow release formulations [47,48], which have a prolonged effect could be evaluated.

Our model also allows us to evaluate the effect of modifying the coverage of the intervention. Treating 80% or more of the dogs would mean that human infections could still be reduced using systemic insecticides that are less effective or have a shorter duration. However previous studies (e.g. those with impregnated collars) have shown that it may be difficult to reach a high coverage in dogs in some ZVL endemic regions [49]. Other strategies such as targeting highly infectious dogs (or ‘superspreaders’) could be more efficient in reducing *L*. *infantum* transmission [44].

Our model assumes that dog, sand fly and human populations are constant, and thus that the insecticide does not affect the sand fly-to-host ratios. We have therefore only evaluated the impact that systemic insecticides would have on sand fly survival, not on sand fly density. Reducing sand fly density could also reduce the risk of *Leishmani*a transmission as shown by Poché et al [32]. The model does not consider other potential source of infection such as synanthropic animals or humans that could play a significant role in transmission and it also assumes a constant risk of infection throughout the year. In some endemic areas *L*. *infantum* vectors are seasonal, for example in more temperate climates [50,51]. In those areas, systemic insecticides with shorter efficacy (e.g. 3 months) may be sufficient to significantly reduce the *L*. *infantum* infections in humans. This scenario was not considered in our model. Neither was the use of repeated treatments (e.g. treating dogs every 3 months), which may be an alternative to reach the efficacy requirements identified in the model. This would, however, increase the cost of the intervention.

Additionally, some aspects of the model could cause bias in the predictions. The parameter that the model output was most sensitive to was the sand fly mortality rate. This parameter is critical since is directly related to the probability of a sand fly surviving 7 days, the extrinsic incubation period of *L*. *infantum* (Fig 2). However, other, lower sand fly mortality rates (μ < 0.42) have been used in modelling of ZVL [12, 23, 25]. This would indicate that our predictions are conservative, i.e. using any of the other reported sand fly mortality rates our model would predict a stronger effect at controlling human infections of *L*. *infantum* using systemic insecticides in dogs.

In ZVL endemic regions (e.g. Brazil) where current control measures are failing to control *L*. *infantum* transmission, the community-wide use of systemic insecticides in dogs could be considered as an alternative or complementary vector control strategy. In this study we did not compare different interventions but previous models using similar multi-compartmental models have shown that the risk of *L*. *infantum* transmission can be significantly reduced by the use of insecticide-impregnated dog collars [16,36]. Both interventions would reduce the number of infected sand flies so similar results can be expected. Compared to the mass-use of insecticide impregnated dog collars, systemic insecticides may be easier to deploy, in particular if oral formulations are used. Nevertheless, a number of operational challenges can be expected. As in other mass-treatment interventions high coverage may be difficult to reach. The systemic insecticides currently used in dogs against fleas and ticks have shown to be save when administered based on weight groups [52–54] but its mass use in dogs has never been tested. Dogs may require repeated treatments and adverse effects may need to be monitored. Similarly, estimating the cost of this new intervention is difficult as there are no systemic insecticides for dogs registered against sand flies. The cost of community-wide use of dog collars in Brazil has been reported at 12 USD/dog assuming one cycle of intervention [38,55], other authors have reported a cost just per collar around 10–15 USD [21]. The cost-effectiveness of the use of systemic insecticides in dogs to control ZVL remains to be proven.

The product requirements identified in our model could guide the development of a new product or the repurposing of systemic insecticides already available so that they can be used as a public health intervention to control ZVL in endemic regions.
