# Correction: Estimating the efficacy of community-wide use of systemic insecticides in dogs to control zoonotic visceral leishmaniasis: A modelling study in a Brazilian scenario

**DOI:** 10.1371/journal.pntd.0007452

**Published:** 2019-05-28

**Authors:** Sonia A. Gomez, Lloyd A. C. Chapman, Erin Dilger, Orin Courtenay, Albert Picado

There is an error in Table 1. The value in line 3 read “Death rate of infected dogs” currently reads 0.003006/day and the reference reads [42]. The correct vales in line 3 "Death rate of infected dogs" should be 0.0018/day and the reference should be [38]. A copy of the correct table is below.

**Table 1:** Parameters in the model and their sources.

Parameter	Definition	Value	Reference
ρ	Proportion of highly infectious dogs	0.17	[15]
i	Incubation rate in dogs	0.005/day	[41]
δ	Death rate in non-infectious dogs	0.0011/day	[15]
δ_i_	Death rate of infected dogs	0.0018/day	[38]
a_D_	Biting rate on dogs	0.333/day	[43]
a_H_	Biting rate on humans	0.125/day	[36]
τ	Latent period of *L*. *infantum* in sand flies	7 days	[39]
μ	Sand fly mortality rate	0.42/day (57%)	[39]
μ_T_	Sand fly mortality rate under treatment	Variable (57–100%)	-
V	Number of sand flies	12000	Fixed
H	Number of humans	1000	Fixed
N	Number of dogs	1000	Fixed
$p_{v}^{hi}$	Probability of a highly infectious dog transmitting to a sand fly	0.39	[15]
$p_{v}^{li}$	Probability of a low-infectious dog transmitting to a sand fly	0.017	[15]
p_D_	Probability of an infected sand fly transmitting to a dog	0.321	[16]
*P_T_* Δ	Proportion of dogs treated with systemic insecticide Decay/day in insecticide efficacy after the administration	Variable (60, 70, 80%) Variable (-0.0001 –-0.05)	- -
